# Self-motion perception is sensitized in vestibular migraine: pathophysiologic and clinical implications

**DOI:** 10.1038/s41598-019-50803-y

**Published:** 2019-10-04

**Authors:** Susan King, Adrian J. Priesol, Shmuel E. Davidi, Daniel M. Merfeld, Farzad Ehtemam, Richard F. Lewis

**Affiliations:** 1 Jenks Vestibular Physiology Laboratory, Massachusetts Eye and Ear Infirmary, Boston, MA USA; 2 Department of Otolaryngology, Harvard Medical School, Boston, MA USA; 3 Department of Otolaryngology, Ohio State University, Columbus, OH USA; 4 Speech and Hearing Biotechnology Program, Harvard Medical School, Boson, MA USA; 5 Department of Neurology, Harvard Medical School, Boston, MA USA

**Keywords:** Sensory processing, Migraine

## Abstract

Vestibular migraine (VM) is the most common cause of spontaneous vertigo but remains poorly understood. We investigated the hypothesis that central vestibular pathways are sensitized in VM by measuring self-motion perceptual thresholds in patients and control subjects and by characterizing the vestibulo-ocular reflex (VOR) and vestibular and headache symptom severity. VM patients were abnormally sensitive to roll tilt, which co-modulates semicircular canal and otolith organ activity, but not to motions that activate the canals or otolith organs in isolation, implying sensitization of canal-otolith integration. When tilt thresholds were considered together with vestibular symptom severity or VOR dynamics, VM patients segregated into two clusters. Thresholds in one cluster correlated positively with symptoms and with the VOR time constant; thresholds in the second cluster were uniformly low and independent of symptoms and the time constant. The VM threshold abnormality showed a frequency-dependence that paralleled the brain stem velocity storage mechanism. These results support a pathogenic model where vestibular symptoms emanate from the vestibular nuclei, which are sensitized by migraine-related brainstem regions and simultaneously suppressed by inhibitory feedback from the cerebellar nodulus and uvula, the site of canal-otolith integration. This conceptual framework elucidates VM pathophysiology and could potentially facilitate its diagnosis and treatment.

## Introduction

Migraine is characterized by recurrent headache but more generally is considered a neurologic disorder of sensitization, as the headaches are characteristically associated with heightened sensitivity to light, sound, and other sensory stimuli^1^. Vestibular symptoms are particularly common in migraine^2^–in addition to enhanced motion sickness susceptibility^3^ many migraineurs experience discrete episodes of vertigo or ataxia that differ from other common migraine auras, as vestibular episodes often occur without a headache and have a highly variable range of durations, lasting from seconds to weeks^4^. These episodic vestibular symptoms caused by migraine, referred to as vestibular migraine (VM), have been estimated to affect 1% of the general population^2^ and to cause about 50% of episodic dizziness in children^5^ and 35% in adults. Indeed, it is now considered the most common cause of episodic vestibular symptoms^6^.

Despite its prevalence and morbidity, VM remains poorly understood. In particular, no physical signs or test abnormalities have been identified that are pathognomonic for VM so its diagnosis is based on criteria defined by an expert panel^7,8^. Further, the mechanisms that relate migraine to vestibular symptoms remain uncertain, although there are numerous potential anatomic substrates given the extensive projections between brainstem regions associated with migraine and the vestibular nuclei (see^9^ for a review). Since peripheral and central sensory systems are sensitized in migraine^10,11^, a reasonable hypothesis is that VM differs from other forms of migraine because vestibular pathways are sensitized in a manner that leads to both enhanced motion sickness susceptibility^12^ and episodic vertigo. Our prior studies^13–15^ suggested that VM patients may be particularly sensitive to the combined modulation of semicircular canal (angular velocity) and otolith (gravito-inertial) vestibular cues produced by roll tilt of the head, and recent studies from other laboratories^16–18^ support the contention that spatial orientation in roll is abnormal in VM.

We therefore measured the frequency-dependence of self-motion perceptual thresholds in VM patients during movements that activated the canals and otolith organs in combination and in isolation, the vestibulo-ocular reflex (VOR), and the severity of dizziness, motion sickness, and migraine headaches. Our results provide evidence for a VM *perceptual biomarker*, since roll tilt thresholds in VM were abnormally reduced in a frequency-dependent pattern. Overall, our findings support a pathogenic mechanism that could explain some of the vestibular symptoms caused by migraine, namely that these symptoms could emanate from the vestibular nuclei, which are simultaneously activated by migraine regions in the brainstem and suppressed by a negative feedback loop through the cerebellar nodulus and uvula (the anatomic locus for canal-otolith integration^19^), where sensitization is compensatory and evidenced by reduced tilt thresholds.

## Results

Data described in *sections 1–3* are from the cohort of subjects who underwent all threshold protocols (12 normal, migraine, and VM patients), and the 8 Meniere’s disease patients who were tested on roll tilt at 0.2 Hz. *Sections 4–5* describe data from a larger (n = 29) VM group whose testing consisted of roll tilt perceptual thresholds at 0.05, VOR testing, and quantification of symptom severity. Patient groups were identified using the standard, accepted diagnostic criteria for migraine^20^, VM^8^ and Meniere’s disease^21^.

### Basic characteristics of the VM and control groups

Symptom severity and demographics of the VM and control groups are summarized in Table 1. The principal observations are that VM patients had significantly greater dizziness as assessed with the Dizziness Handicap Inventory (DHI^22^) than the migraine or normal groups, and motion sickness susceptibility (MSSQ^23^) was significantly increased in VM patients compared to migraine or normal controls. Headache severity (assessed with the Headache Impact Test^24^) was more pronounced in the migraine and VM groups than the normal subjects, as expected, with no significant difference between the migraine and VM groups. Headache severity was uncorrelated with vestibular symptoms (DHI, MSSQ) in normal and migraine subjects, but it did correlate with dizziness (DHI) in VM patients (Pearson R test p = 0.02). Anxiety assessed with the Beck Anxiety inventory^25^ did not differ significantly between the VM, migraine, and normal control groups. The VM, migraine, and normal groups did not differ in age, and the migraine and VM groups were gender-matched while the normal group was evenly divided between genders.

**Table 1:** Clinical characteristics of the vestibular migraine, migraine, and normal groups.

Clinical measure	Vestibular migraine (VM)	Migraine (M)	Normal (N)	Statistical comparisons
*DHI	26.7 +/− 6.5	0.2 +/− 0.2	0.8 +/− 0.9	M-W: p < 0.001 for VM-M, p = 0.002 for VM-N
*MSSQ	32.2 +/− 4.5	10.4 +/− 3.4	13.9 +/− 4.9	t-test: p < 0.001 for VM-M, p = 0.02 for VM-N
HIT	56.7 +/− 2.3	53.9 +/− 4.2	41.7 +/− 1.2	t-test: p = 0.6 for VM-M
BA	13.0 +/− 3.2	8.0 +/− 1.8	5.6 +/− 2.3	t-test: >0.1 for VM-M and VM-N
Age	36.5 +/− 2.7	34.0 +/− 1.1	38.1 +/− 3.1	ANOVA p = 0.39
Sex (F:M)	11:1	10:2	6:6	

Standard questionnaires were used to quantify the severity of dizziness (DHI^22^), motion sickness susceptibility (MSSQ^23^), headache (HIT^24^), and anxiety (BA^25^), which are shown as means +/− one SEM for the 12 subjects in each category (as is age). Gender distribution is shown as female:male. Statistical tests are Mann-Whitney (M-W), t-tests, and one-way analysis of variance (ANOVA).

### Roll tilt perceptual thresholds are reduced in VM at mid and low frequencies

**Figure 1:**
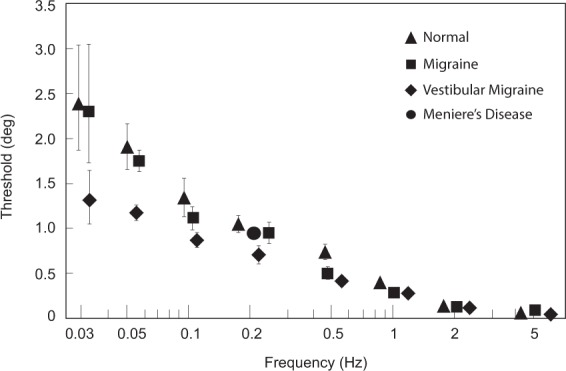
Roll tilt perceptual thresholds (geometric means+/− one SE) in degrees for the four subject groups, plotted against motion frequency. VM, migraine, and normal subjects were tested at all frequencies and Meniere’s Disease patients were tested at 0.2 Hz. Icons are triangles (normal), squares (migraine), diamond (VM), and circle (Meniere’s disease). For all data points, n = 12 for VM, migraine, and normal groups (except for 0.03 Hz where the n is 4 for these three groups) and n = 8 for Meniere’s patients. Some error bars are smaller than the associated icons and therefore are not visible. Less data is available for 0.03 Hz tilt because most subjects lacked the attention to perform this task adequately (as each trial lasted 33.3 s).

The geometric means of the roll tilt perceptual thresholds for the four subject groups, recorded using standard motion stimuli^26^ and psychometric methods^27^, are shown in Fig. 1. All groups showed a similar frequency-dependence whereby displacement thresholds (the smallest position change that was accurately perceived as rightward or leftward) were smallest at the higher frequencies and larger at the lower frequencies – consistent with earlier normative data^28^. Roll tilt thresholds depended significantly on both the subject group (VM, migraine, normal, ANOVA p < 0.001) and on the motion frequency (ANOVA p < 0.001). Furthermore, in the mid-to-low frequency range (0.03, 0.05, 0.1, and 0.2 Hz) VM thresholds were significantly lower than normal subjects (t-test: p = 0.001) and migraine patients (t-test: p = 0.001), while migraine and normal subjects did not differ (t-test: p = 0.81). Over the higher frequency range (1.0 Hz and above) thresholds in the three groups converged and did not differ significantly (ANOVA p = 0.1; t-tests between groups all > 0.05). Migraine patients had thresholds that were equivalent to normal controls except for 0.1 and 0.5 Hz, where their thresholds fell between the normal and VM groups but did not differ significantly from normal (t-tests p = 0.1 for 0.5 Hz, p = 0.4 for 0.1 Hz). Patients with Meniere’s disease, who had recurrent vertigo not due to migraine, had normal thresholds at 0.2 Hz, a frequency where VM thresholds were significantly lower than the control groups. The conclusion from these data is that *roll tilt thresholds are abnormally small in VM patients over the mid to low-frequency range*, *but this was not due to migraine (without episodic vertigo) or episodic vertigo (without migraine)*.

### Reduced roll tilt thresholds in VM reflect abnormal canal-otolith integration

**Figure 2:**
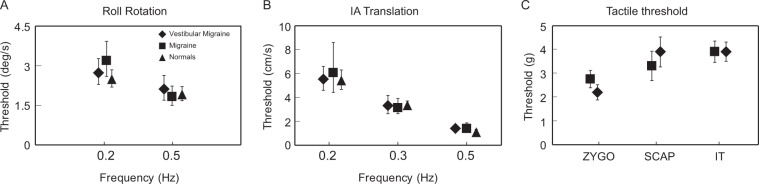
Perceptual thresholds in the VM and control groups for (**A**) roll rotation about an earth-vertical axis; (**B**) inter-aural (IA) translation along an earth-horizontal axis; and (**C**) pressure thresholds measured with Semmes-Weinstein monofilaments^72^ at three locations (zygomatic arch, scapula, and iliac tuberosity, with the left and right-sided measurements averaged for each subject and location). Tactile thresholds were measured only in the VM and migraine groups since tactile sensitization related to allodynia was a potential concern in the two migraine populations but not in the normal control subjects.

Roll tilt modulates activity in the vertical canals, the graviceptive organs (primarily the utricles for the small tilt angles we employed), and the tactile cues applied to the head and trunk. We therefore tested each of these three sensory components in isolation to determine if they were responsible for the threshold reduction in VM during roll tilt. Figure 2 shows perceptual thresholds for roll rotation while supine (vertical canals), inter-aural (IA) translation (utricles), and tactile thresholds over 3 dermatomes on each body side, and in all cases thresholds in VM and control subjects were indistinguishable (roll rotation: 2-way ANOVA, p = 0.79 for group, 0.1 for frequency, and 0.9 for interaction; IA translation: 2-way ANOVA, p = 0.51 for group, p < 0.001 for frequency, and p = 0.97 for interaction; tactile Mann-Whitney p = 0.34). In particular, below 0.5 Hz roll tilt thresholds were abnormally low in VM but roll rotation and IA translation thresholds were normal. Together these results indicate that the roll tilt threshold reduction in VM is not due to sensitization of canal or otolith signals in isolation, nor does it reflect increased tactile sensitivity. Since prior work in normal subjects (e.g.^28^) demonstrated that roll tilt thresholds at mid and low frequencies require central integration of canal and otolith cues, *abnormal roll tilt thresholds in VM appear to result from changes in the synthesis of canal and otolith inputs in the brain*.

### Roll tilt thresholds in VM are related to vestibular symptoms

**Figure 3:**
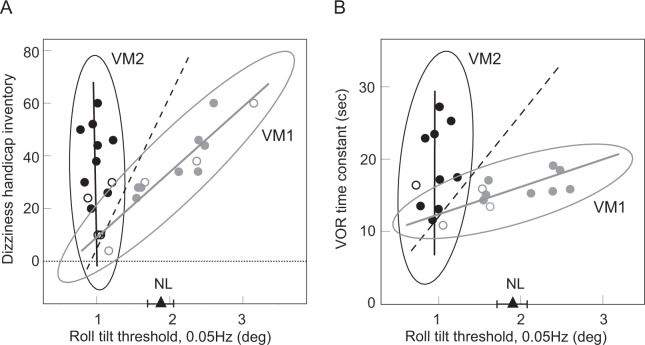
Correlations between perceptual thresholds and other vestibular characteristics. (**A**) Dizziness symptom severity (DHI score) vs. 0.05 Hz roll tilt thresholds. The triangle shows the mean of the normal 0.05 Hz roll tilt threshold+/− one SE. The black dashed line (described in the text) segregates VM patients into two subsets, VM1 patients (grey icons) clustered about the grey regression grey line, and VM2 patients (black icons) clustered about the black regression line. DHI is scored from 0 (no dizziness) to 100. (**B**) VOR time constant vs. 0.05 Hz roll tilt thresholds. VM patients were separated into VM1 (grey icons and regression line) and VM2 (black icons and regression line) as in Fig. 3A. For both 3 A and 3B, 95% confidence ellipses are shown for VM1 and VM2, and filled icons are patients with data in both 3 A and 3B while open icons are patients with data in either 3 A or 3B.

The relationship between vestibular symptom severity (DHI score) and roll tilt thresholds at 0.05 Hz is shown in Fig. 3A for the expanded VM population tested at this frequency. The data appear to be distributed in a pattern that approximates a tilted ‘V’ with the two limbs converging at the lower left corner of the plot. To facilitate analysis of the data we considered the two limbs separately, dividing patients into the VM1 (grey icons) and VM2 (black icons) clusters (Fig. 3A) with an intuitive method based on *visual inspection* of the plot, and a *soft clustering* method which required no assumptions about the data’s distribution except that it was divisible into two subsets: (i) for the *visual approach*, we calculated the (black dashed) line that connected the average (DHI, threshold) values for all VM patients with the average values for the three data points located in the lower left corner of the plot where the two limbs of the ‘V’ converge. This line was used to separate the VM patients into VM1 and VM2 clusters and the VM1 and VM2 data were each fit with a linear regression (grey and black lines, respectively) and with 95% confidence ellipses (Fig. 3A); and (ii) to verify this visual classification, we also segregated the VM data using *soft clustering*, in which the probability of the patient belonging to each cluster is calculated^29^, with points at the edge of a cluster having lower probabilities of membership to that cluster^30,31^. As detailed in the methods, the results of the visual and soft clustering approaches were very similar–ROC AUC analysis comparing these two classification methods yielded a value of 0.93.

*The principal difference between VM1 and VM2 was the relationship between vestibular symptom severity (DHI) and roll tilt threshold*. Using the visual classification as our standard approach, *VM1 showed a positive correlation between tilt threshold and symptom severity* (Pearson R for DHI and tilt threshold, R = 0.94, p < 0.001) and therefore a *negative correlation between tilt sensitivity and dizziness*. In contrast, *VM2 patients had DHI values that varied independently of their tilt thresholds*. Furthermore, tilt thresholds in VM2 were uniformly low (less than normal) but thresholds in VM1 were more widely distributed and included values above, within, and below the normal range. Comparison of other VM1 and VM2 characteristics (Table 2) indicates that they were equivalent except for the roll tilt threshold, which was lower in VM2, and the motion sickness susceptibility score, which was higher in VM1. Otherwise, VM1 and VM2 s shared equivalent dizziness and headache symptom severity and equivalent ages and gender distributions.

**Table 2:** Characteristics of the two vestibular migraine subgroups (VM1, VM2) identified in Fig. 3A (DHI versus tilt threshold).

Clinical measure	VM1	VM2	Statistical comparisons
DHI	32.1 +/− 4.6	31.7 +/− 4.6	t-test: p = 0.47
*MSSQ	30.0 +/− 3.1	19.7 +/− 3.4	t-test: p = 0.02
HIT	55.2 +/− 1.9	56.0 +/− 2.1	t-test: p = 0.40
Age (years)	38.9 +/− 3.3	34.0 +/− 3.4	t-test: p = 0.17
Sex (F:M)	9 F:3 M	11 F:2 M	Rank test: p = 0.5
*Roll tilt threshold (deg)	1.94 +/− 0.17	1.01 +/− 0.04	M-W RST: p < 0.001

Abbreviations are defined in the Table 1 legend; roll tilt thresholds were measured at 0.05 Hz. All values are shown as means +/− one SEM. M-W is Mann-Whitney and M-W RST is rank sum test. Asterisks indicate clinical measures that differ between VM1 and VM2.

### Roll tilt thresholds in VM are related to VOR dynamics

Velocity storage is the brainstem mechanism that mediates the dynamic and spatial characteristics of vestibular behaviors^32,33^ and in particular it determines the time constant (Tc) of the VOR. Similar to the DHI-threshold data shown in Fig. 3A, the relationship between the VOR Tc and roll tilt thresholds (Fig. 3B) also approximated a tilted ‘V’ with the two limbs converging at the lower left corner of the plot. We employed the same two approaches (visual and soft clustering), therefore, to separate the two limbs of the Tc-threshold distribution into VM1 (grey icons) and VM2 (black icons) clusters. Again, as detailed in the methods the results of the visual and soft clustering approaches were very similar with ROC value of 0.99, and all VM patients with DHI and VOR Tc data available (filled icons in Fig. 3A,B) independently received the same label (VM1 or VM2) for both the DHI-threshold and Tc-threshold analyses. Paralleling the DHI-threshold data described above, the *principal difference between VM1 and VM2 for the Tc-threshold data was the relationship between the VOR time constant and roll tilt threshold*, *asVM1 patients showed a positive correlation between the VOR time constant and tilt threshold* (Fig. 3B, grey data points and line, Pearson R, R = 0.76. p = 0.01) while *VM2 patients had Tc values that varied independently of the uniformly low tilt thresholds* (Fig. 3B, black data points and line). VM1 and VM2 patients identified with the Tc-threshold analysis (Table 3) were otherwise comparable except for the roll tilt threshold, which was lower in VM2 (note that some patients had only DHI or Tc data available, open icons in Fig. 3, so values in Tables 2 and 3 differ).

**Table 3:** Characteristics of the two vestibular migraine subgroups (VM1, VM2) identified in Fig. 3B (VOR time constant versus tilt threshold).

Clinical measure	VM1	VM2	Statistical comparisons
VOR time constant (sec)	15.5 +/− 0.7	18.8 +/− 1.7	M-W RST: p = 0.2
MSSQ	26.4 +/− 4.6	22.7 +/− 5.4	t-test: p = 0.6
HIT	56.9 +/− 2.2	51 +/− 5.9	M-W RST: p = 0.7
Age (years)	39.4 +/− 2.7	34.5 +/− 4.5	t-test: p = 0.35
Sex (F:M)	9 F:2 M	9 F:1 M	Rank test: p = 0.6
*Roll tilt threshold (deg)	1.83 +/− 0.15	0.97 +/− 0.5	M-W RST: p < 0.001

Abbreviations are defined in the legend for Table 2.

## Discussion

Our primary findings are that roll tilt perceptual thresholds in VM: i) are abnormally low for mid and low frequency motion stimuli; ii) are not due to migraine (when it is unassociated with vestibular symptoms), or due to vestibular symptoms (when they are not due to migraine); iii) reflect abnormal integration of canal and otolith information in the brain; iv) have a ‘V’ shaped distribution with respect to the severity of vestibular symptoms, with data clustered about one leg displaying a positive correlation between tilt threshold and symptom severity and data clustered about the other leg displaying threshold-independent symptoms but uniformly low tilt thresholds; and v) have a very similar ‘V’ shaped distribution with respect to velocity storage (VOR time constant). These results are discussed below in terms of their potential diagnostic, pathophysiologic, and therapeutic implications.

### Diagnostic implications

VM is the most common cause of episodic vestibular symptoms^6^ but lacks any pathognomonic finding and is diagnosed using criteria determined by an expert panel^7,8^. Identifying an approach that segregates VM patients from vestibular patients whose symptoms are not due to migraine would therefore be of great clinical value. Our results show that for populations of patients, VM differs from vestibular (without migraine) patients, migraine (without vestibular) patients, and normal subjects with respect to mid and low frequency roll tilt perceptual thresholds. The mechanisms responsible for these findings are considered below, but from a clinical perspective these results could provide a *biomarker* that allows individual patients to be classified as VM or non-VM based on their roll tilt threshold when considered in tandem with their DHI and/or VOR time constant. To determine the sensitivity and specificity of this approach, substantially larger patient populations must be studied to more accurately define the VM confidence ellipses and to determine the data structure for vestibular patients whose symptoms are not due to migraine. Ultimately a multivariate analysis that includes the low frequency roll tilt thresholds, DHI, and VOR time constant, and other perceptual (e.g., z-axis translation thresholds^34^) or VOR (e.g., response variability) measures may be a way to most accurately separate VM and non-VM vestibular patients.

Such a multivariate analysis may be particularly helpful because there are a plethora of possible interactions between migraine and the vestibular system^9^, so a *heterogeneous set of mechanisms almost certainly contributes to the vestibular symptoms of VM*. For example, we followed the current VM classification criteria^7,8^ in this study, so patients with evidence of a peripheral vestibular damage were excluded. Migraine is epidemiologically linked to Meniere’s disease^35^, however, some migraine patients develop evidence of labyrinthine damage^36^, and animal models of migraine show evidence of biochemical changes in the labyrinth^37^, implying that vestibular dysfunction in VM may have a peripheral component in some patients^38^. While the current VM diagnostic criteria allowed us to focus on central mechanisms without confounding peripheral effects that could potentially mask central changes, diagnostic approaches for VM must ultimately consider potential central (infratentorial, supratentorial) and peripheral pathophysiologic changes.

### Pathophysiologic implications

To facilitate interpretation of our results, we first summarize the relevant concepts of central vestibular processing (reviewed in^33^); then consider the implications of our findings when examined within this conceptual framework; and finally provide a hypothesis for mechanisms that could contribute to the vestibular symptoms of VM that is consistent with our results and with known vestibular physiology and migraine pathophysiology.

#### Overview of relevant central vestibular processing

**Figure 4:**
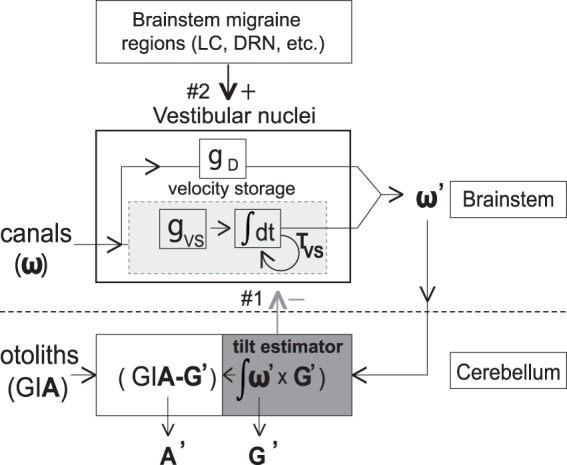
Schematic diagram showing the processing of vestibular signals in the brainstem and cerebellum. Physical parameters sensed by the canals and otoliths are angular velocity (**w**) and gravito-inertial acceleration (GI**A**) respectively, and central estimates are angular velocity (**w’**), orientation of gravity (**G’**), and linear acceleration (**A’)**. Canal inputs are processed through a direct pathway with gain g_D_ and an indirect (velocity storage, light grey box) pathway which functions as a leaky integrator with gain g_VS_ and time constant T_VS_^33,52^. The cerebellar NU (‘tilt estimator,’ dark grey box) inhibits velocity storage (grey arrow #1) by shortening the time constant of the velocity storage integrator. Brainstem regions associated with migraine (e.g., LC = locus coeruleus; DRN = dorsal raphe nucleus) also project to the vestibular nuclei (black arrow #2)–LC projections are noradrenergic^53^ and DRN projections are serotonergic^54^. Letters in bold are three-dimensional vectors.

As shown schematically in Fig. 4, the labyrinth transduces high-pass filtered angular velocity in three dimensions (**w**, canals) and the vector sum of gravity and linear acceleration (GI**A**, otoliths). The brain estimates angular velocity (**w’**) by combining the direct signal from the canal afferents (g_D_ pathway) with a temporal integration of the canal afferent signal (via *velocity storage*, with a gain g_VS_ and a time constant T_VS_). Velocity storage (light grey box, Fig. 4) is generated in the commissural fibers between the medial and superior vestibular nuclei and hence can be considered a marker of vestibular nuclear activity^39,40^. It is responsible for the dynamics of VOR and perceptual responses induced by head rotation (primarily in yaw)^41,42^. The nodulus and uvula of the cerebellum (NU) are the location of the ‘tilt estimator” (dark grey box, Fig. 4) where canal and otolith inputs are first synthesized^43^. Specifically, the NU integrates the off-vertical component of the angular velocity signal to update the estimated orientation of gravity **G’**^44,45^, and **G’** is subtracted from the GI**A** sensed by the otoliths to estimate **A’** (linear acceleration)^46,47^. The NU tilt estimator has GABAergic inhibitory projection to the vestibular nuclei^48^ (Fig. 4, grey arrow #1), including the velocity storage network, as NU ablation lengthens^49^ and stimulation shortens^50^ the VOR Tc.

#### Evidence that reduced VM tilt thresholds reflect sensitization of the cerebellar NU

**Figure 5:**
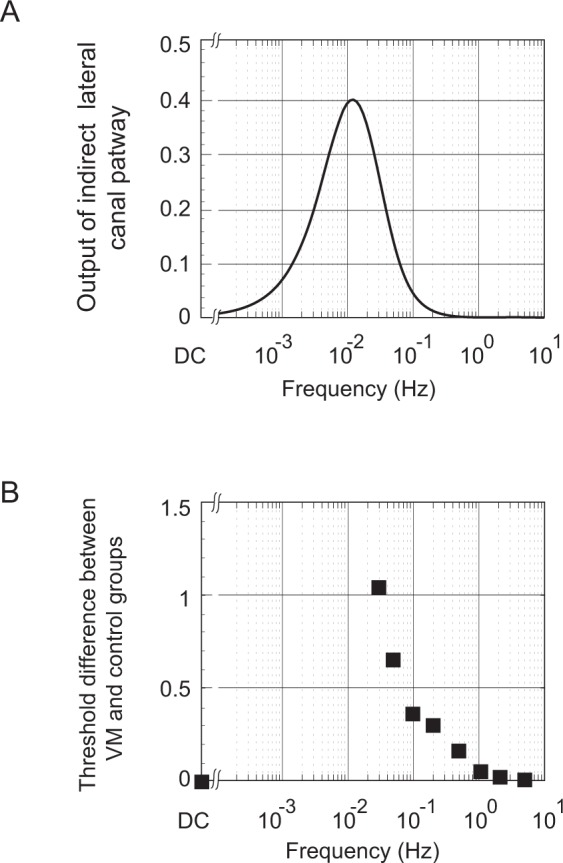
Comparison of frequency-dependence of velocity storage and vestibular migraine thresholds. (**A**) Frequency-dependence of the “indirect” velocity storage pathway (simulated with the Raphan and Strum model^52^) using the T_VS_ measured in VM patients (20.9 s) and an arbitrary gain value. The combination of the canal high-pass filter and the velocity storage low-pass filter in series yields an output that peaks between the canal and velocity storage cut-off frequencies (approximately 0.03 Hz and 0.01 Hz respectively). (**B**) Threshold abnormality in VM patients to roll tilt stimuli (VM threshold – [non-VM] threshold) for each frequency, using geometric means, including the “quasi-static DC” results^13,28^.

Three types of evidence suggest that the NU is sensitized in VM patients – i) *VM thresholds were reduced when canal and otolith cues co-modulated* (e.g., during tilt, Fig. 1) but *not when either the canals or otoliths modulated in isolation* (Fig. 2). Since the NU is the location of canal-otolith integration, the reduction of tilt but not rotational or translational thresholds in VM suggests that central sensitization localizes at least in part to the NU; ii) if the NU is sensitized in VM, a *positive correlation is predicted between tilt thresholds and the VOR time constant* because of the inhibitory projection (Fig. 4, *grey arrow #1*) from the NU (where tilt is calculated) to velocity storage in the brainstem (which sets the VOR Tc). Specifically, NU sensitization (reflected by lowered tilt thresholds) should result in increased inhibition of velocity storage (reflected by lowered Tc). Indeed, one cluster of VM patients (Fig. 3B, VM1 = *grey icons and line*) showed this predicted correlation, as did the non-VM subjects (data not shown). In contrast, the second VM cluster (Fig. 3B, VM2 = *black icons and line*) had uniformly low tilt thresholds that were independent of the VOR Tc (discussed below); and iii) since the NU projects to and receives reciprocal projections from vestibular nuclei^48,51^ where velocity storage resides (Fig. 4), if reduced thresholds indicate sensitization of the NU then the *frequency-dependence of the threshold abnormality should mirror that of velocity storage*^52^. Figure 5 compares the frequency-dependence of the VM threshold abnormality and velocity storage and demonstrates their close similarity (this figure includes “pseudo-static” DC^28^ results from^13^), supporting our contention that *tilt threshold abnormalities in VM localize to the NU because they are closely related to velocity storage (which is inhibited by the NU)*.

#### Evidence that the vestibular nuclei contribute to the symptoms of VM and are modulated by (inhibitory) cerebellar and (excitatory) brainstem projections

The relationship between tilt thresholds and the VOR Tc in VM patients (Fig. 3B) is structurally the same as the relationship between tilt thresholds and vestibular symptom severity (DHI, Fig. 3A). Both the threshold-Tc and threshold-DHI plots have two clusters of data, one grouped about a positive regression (grey icons and lines) and one grouped about a line that is nearly parallel to the y-axis with uniformly low thresholds (black icons and lines). Since the VOR Tc is a well characterized measure of *vestibular nuclear activity* (velocity storage in particular), one logical explanation for the similarity in plots 3 A and 3B is that vestibular symptoms in VM are generated, at least partially, within the brainstem vestibular nuclei in a manner that correlates with the processing of the VOR Tc. Like the threshold-Tc data (Fig. 3B), the positive correlation between tilt thresholds and vestibular symptoms in Fig. 3A (*grey regression line*) likely reflects the *inhibitory* projections^48^ from the cerebellar NU to the vestibular nuclei (Fig. 4, *grey arrow #1*). Further, the vestibular nuclei receive multiple projections from brainstem regions associated with migraine initiation and propagation, including the trigeminal nuclei, locus coeruleus, and dorsal raphe nucleus^53–55^. We propose, therefore that both the vestibular symptoms of VM and the VOR Tc can also be modified independent of NU activity/tilt thresholds (*black regression lines* in Fig. 3A,B) because of the effects of *excitatory* projections from these migraine-related brainstem regions to the vestibular nuclei (Fig. 4, *black arrow #2*).

The VM patients have a ‘V’ like distribution when tilt thresholds are plotted against dizziness severity or the VOR Tc, with the VM1 and VM2 legs merging in the low threshold–low DHI/Tc corner of the plots (Fig. 3). This pattern suggests that VM1 and VM2 could be distinct but overlapping phenotypes or that they could represent two components of a single VM phenotype. Our pathogenic model, discussed above, supports the single phenotype explanation with the VM1 and VM2 clusters representing a continuum of patient states that are determined by the relative contributions of inhibitory (cerebellar) and excitatory (brainstem) projections to the vestibular nuclei. Since patients with less severe vestibular symptoms and/or short VOR Tc always have low tilt thresholds, feedback inhibition supplied by the cerebellar NU to the vestibular nuclei (Fig. 4, grey arrow #1) may be sensitized as an early response to the aberrant vestibular nuclei activation produced by migraine (as evidenced by the positive correlation in VM1 between DHI/Tc and tilt thresholds, Fig. 3, grey lines). This inhibitory feedback may be adequate in some patients, namely those with low thresholds but also low DHI or VOR Tc values (bottom left corner of Fig. 3A,B). Cerebellar-mediated inhibition presumably saturates when the NU has reached maximum sensitization, associated with the lowest possible roll tilt thresholds, and then additional stimulation of the vestibular nuclei by brainstem migraine regions (Fig. 4, black arrow #2) increases the DHI and VOR Tc without influencing the already low threshold (VM2 in Fig. 3A,B, black lines).

Two other aspects of the data distributions shown in Fig. 3 warrant further discussion. Firstly, it is evident that, unlike most of the VM patients who have low tilt thresholds, some VM1 patients have tilt thresholds that are in the high-normal range. The exact explanation for this result is not known but presumably the mechanisms underlying vestibular symptoms in migraine are heterogeneous and can result in a distribution of tilt thresholds that are independent of the model described above. We suggest, therefore, that high-normal tilt thresholds in some VM patients reflect both the inherent variability associated with this disorder and suboptimal engagement of the inhibitory cerebellar feedback loop through the NU that we propose is responsible for threshold reduction. Secondly, our results are qualitatively consistent with prior work that suggested the severity of vestibular symptoms in VM (e.g., motion sickness) correlate with the length of the VOR time constant^12^. In particular, comparison of the VM1 clusters in Fig. 3A,B indicates that higher tilt thresholds are associated with both longer time constants and higher DHI scores, implying that longer time constants correlate with greater vestibular symptomatology. While this correlation was not statistically significant in our relatively small VM data set (as it was in the much larger patient population that was previously described^12^), the general pattern is recapitulated by our results.

#### This model provides a potential explanation for the relationship between vestibular symptoms and migraine

The vestibular nuclei are a node in a negative feedback loop (Fig. 4), as they project to (and could therefore sensitize) the NU, which then suppresses vestibular nuclear activity through reciprocal inhibitory projections. This feedback loop contributes to the brain’s ability to generate accurate motion and orientation estimates and may play a fundamental role in the generation of motion sickness^56^. Persistent dysfunction of this neural circuit in VM could therefore contribute to the characteristic increase in inter-ictal motion sickness susceptibility^3,12^. This neural circuit can oscillate when the NU is damaged and it has been suggested that it is inherently unstable and is stabilized by NU inhibition^50^. In VM patients, a migraine episode could sensitize the vestibular nuclei to the extent that NU suppression is no longer adequate to maintain stability, particularly when NU activity is modulated by changing head orientation, resulting in ictal episodes of vestibular dysfunction that are temporally linked to migraines and induced by head tilts (e.g., are “positional”), both of which are common in VM^4,38^.

#### Correlations with imaging and other behavioral studies

Our model is supported by the one ictal functional imaging study of VM patients^57^ which showed prominent activation of the cerebellum and brainstem during vestibular episodes. Interestingly, the primary anatomic changes in VM appear to be supratentorial, and these changes in the thalamus^58^ and cerebral cortex^59^ may be due to primary migraine-related dysfunction in these brain regions or could in part reflect their role as targets for infratentorial projections. More generally, symptoms of VM are almost certainly heterogeneous in origin and most likely are generated by mechanisms that localize to both infra- and supratentorial^60^ brain structures and possibly to the vestibular labyrinth as well. *Only infratentorial mechanisms were investigated in our study so our findings do not preclude significant contributions from other brain regions or from the inner ear*.

### Therapeutic implications

There are no placebo-controlled prospective clinical trials that assess the efficacy of different pharmacologic treatments for VM^61^ and there is no rationale to guide the choice of medication for individual patients. Clearly, placebo-controlled prospective clinical trials are necessary to develop evidence-based guidelines for VM medical therapy, and we suggest that our results (and perceptual threshold testing prior to therapy) could help provide a framework for future clinical trials. Specifically, vestibular symptoms in VM patients with relatively high thresholds (e.g., falling near the VM1 grey cluster on Fig. 3A) may respond best to drugs that activate GABA receptors, since these would increase inhibition of the brainstem vestibular nuclei and potentially suppress ictal and inter-ictal vestibular symptoms. Potential drugs would include those that increase synaptic GABA such as tiagabine^62^ or the aminopyridines that augment GABA release by cerebellar Purkinje cells^63^. This approach has not been tried but positive results from a trial of acetazolamide^64^, which augments GABAergic neuronal activity as one of its properties, offer support for the contention that increasing suppression of brainstem vestibular nuclei may benefit VM patients. Conversely, patients with low tilt thresholds (falling near the VM2 black cluster on Fig. 3A) may have saturated GABAergic inhibition of the vestibular nuclei and may therefore respond best to medications that affect other neurotransmitters such as beta blockers^61^.

While their effects on vestibular symptoms due to migraine have not yet been studied, drugs that inhibit the action of calcitonin gene-related peptide (CGRP) in the trigeminal ganglion such as erenumab (reviewed in^65^) could be important in preventing or aborting vertigo episodes in patients with VM. In particular, since migraine-related sensitization of the trigeminal nuclei appears to affect the sensitivity of structures that receive trigeminal nuclear projections (e.g.^66^, for the visual system), it is possible that the well-characterized trigeminal nuclei → vestibular nuclei projections in the brainstem^55^ could contribute to vestibular sensitization in migraine patients who develop VM. Attenuating the effects of this pathway with drugs the inhibit CGPR activity in the trigeminal ganglion could potentially minimize trigeminal-mediated sensitization of the vestibular nuclei and thereby stabilize the central vestibular feedback loop (Fig. 4).

## Materials and Methods

All subjects provided informed consent in accordance with the Declaration of Helsinki, and all studies were approved by the Massachusetts Eye and Ear Infirmary (MEEI) IRB.

### Experimental design

The objective of this study was to measure self-motion perceptual thresholds and other information about vestibular function and symptoms in patients with VM and in the appropriate control groups, with the goal of elucidating the pathophysiology of VM. Blinding was not employed for the vestibular threshold tests since the threshold values were calculated using an automated “expert advisor” that chose the motion parameters and converged on the threshold in a manner that was independent of the experimenter^67^. For tactile threshold tests, the experimenter was blinded to the subject category. Test order was randomized as described below. *A priori* power calculations were performed using data from our prior preliminary study^13^, with an α = 0.05 and a power of 0.8. The thresholds during roll tilt at 0.01 Hz were 0.55 for VM and 1.4 for migraine, with standard deviations of 0.8 and 0.7, so 8 subjects in each group were calculated as the minimum to achieve the desired power.

### Subject selection/characterization

(a) **VM** patients were culled from the otoneurology clinics at MEEI. They met the currently accepted diagnostic criteria for definite VM^8^ which includes a history of episodic vestibular symptoms that are temporally associated with headaches that meet the International Headache Society (IHS) criteria for migraine^20^; and an absence of any other neurologic or otologic dysfunction responsible for the vestibular symptoms, as assessed with history, physical examination, audiogram, MRI of the brain, and standard clinical rotational testing^68^. 12 VM patients were tested on the full range of perceptual tests, while an additional 17 VM patients were tested only on roll tilt at 0.05 Hz, since that motion profile was identified in the initial cohort of 12 subjects as one that showed a large difference between VM and control subjects and was feasible to test in this larger population (details below); (b) **Migraine** (n = 12) patients met the IHS criteria for migraine with or without aura, lacked a history of any vestibular symptoms (other than motion sickness), lacked evidence of any other neurologic or otologic dysfunction as described above, and had normal brain MRI and rotational testing; (c) **Normal** (n = 12) subjects similarly had no evidence of neurologic or otologic disease, no history of migraine or vestibular symptoms, and had normal rotational testing; and d) **Meniere’s Disease** (n = 8) patients who met diagnostic criteria for definite Meniere’s Disease^21^, had episodic vertigo but no migraine history, and had no evidence of permanent peripheral vestibular damage as assessed with rotational testing and cervical vestibular evoked myogenic potentials.

### Other patient features

No migraine or VM patient was on prophylactic migraine medication and all tests were performed during inter-ictal periods and followed the most recent migraine or vestibular episode by at least two weeks. The VM, migraine, and normal groups were age-matched and the VM and migraine groups were sex-matched. The normal control group did not share the female preponderance of the two migraine groups but a large recent study of 105 healthy asymptomatic subjects found no sex-related differences in self-motion perceptual thresholds for any of the five motion stimuli tested^69^, including roll tilt thresholds at both 1 Hz and 0.2 Hz.

### Non-threshold data

All subjects filled out standard questionnaires to quantify the severity of their dizziness (Dizziness Handicap Inventory, DHI^22^), motion sickness susceptibility (MSSQ using the Golding revised questionnaire^23^), headache severity (Headache Impact Test, HIT^24^) and anxiety (Beck Anxiety Inventory^25^). Yaw-axis VOR responses were characterized with standard low-frequency (0.01 to 1.0 Hz) sinusoidal testing which yielded phase, gain, and bias values for each frequency, and these were used to calculate the overall VOR time, gain, and bias constants^68,70^ for each subject.

### Threshold testing

Three separate test sessions were used to obtain the complete perceptual threshold data set for each subject. Each session lasted a maximum of three hours with breaks provided at a regular schedule to minimize fatigue. The testing order was randomized to eliminate possible order effects and each of the three test sessions were separated by a minimum of one week.

#### Perceptual task

Self-motion perceptual thresholds were determined using a standard forced-choice, one-interval, direction-discrimination method as used previously in our and other labs for numerous vestibular threshold studies (e.g.^26^). Briefly, after each motion the subjected responded by pressing one of two buttons to indicate if they perceived the motion towards their right or left, and if they were unsure they were required to guess. An adaptive three-down, one-up protocol was used (magnitude of motion reduced after three correct responses and increased after one incorrect response), the direction of motion was randomized, and the test terminated when the coefficient of variation of the fitted threshold parameter reached a value of less than 0.2. A psychometric function was fit to these binary response data using published methods^71^ and the width (sigma) of fitted psychometric function was defined as the threshold^27^ which is the stimulus magnitude at which 84% of responses are predicted to be correct.

#### Motion profiles

With the exception of “pseudo-static” roll tilt (see below), all motions were based on a single-cycle sinusoidal acceleration yielding a unidirectional bell-shaped velocity profile^26^. The frequency of the motion was defined as the inverse of the period and ranged from 0.03 Hz (period of 33.3 sec) to 5.0 Hz (period of 0.2 sec). It was not feasible to test subjects at frequencies below 0.03 Hz because the long duration of each trial impaired attention and precluded acquisition of an adequate data set. Each type of motion (roll tilt while upright about a naso-occipital, earth-horizontal axis centered between the ears; roll rotation while supine about an earth-vertical, naso-occipital axis centered between the ears), and inter-aural (IA) translation while upright along an earth-horizontal axis) was tested in separate blocks. Static (DC) thresholds (in Fig. 5) were provided for the VM, migraine, and normal groups from our prior study^13^–they were estimated using “pseudo-static” roll tilt^28^, a method where subjects are tilted at very low angular velocities below the canal threshold.

#### Tactile thresholds

These were determined in 3 regions on each side of the body including the zygomatic arch, scapula, and ischial tuberosity using standard Semmes-Weinstein monofilaments^72^.

### Categorizing VM patients into two subsets

**Figure 6:**
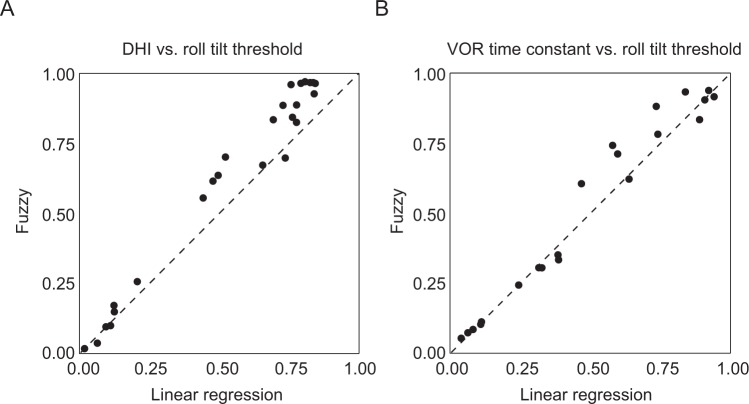
Comparison of vestibular migraine subgroup categorization methods. (**A**) Membership probabilities of belonging to the black cluster for all subjects in the DHI vs. roll tilt threshold plot (Fig. 3A). The x axis shows the probabilities calculated using the intuitive, visual approach based on a *linear regression* separating the data into two groups (dashed black line in Fig. 3A) and the y axis shows the probabilities calculated with the *fuzzy clustering* method. (**B**) Membership probabilities of belonging to the black cluster for all subjects in the VOR time constant vs. roll tilt threshold plot (Fig. 3B). Perfect agreement between the classification probabilities produced by the two methods would result in data points falling along the y = x (dashed) line.

As outlined above, the VM patients appeared to segregate into two clusters when their low-frequency roll tilt thresholds where plotted against DHI or the VOR time constant. We used two methods to separate the VM patients into clusters – i) a *visual*, *intuitive* method was used, since the line connecting the mean of all data points on these plots with the values where the two sets converge in the lower left corner of the plots (dashed black lines in Fig. 3A,B) divided the patients into two visually-distinct groups; and ii) to avoid any subjective influences on patient categorization, we also used a commonly employed *fuzzy c-means clustering* algorithm^29,30^ where membership probabilities for the two clusters are assigned in a way that minimizes distances to each centroid (e.g., minimizing ∑_i_∑_j_ w_i_ u_ij_^m^ d_ij_ where w_i_ is the weight assigned to observation *i*, u_ij_ is the membership of observation *i* in cluster *j*, *m* is the degree of fuzzification^30^, and d_ij_ is the distance between observation *i* and the centroid of cluster *j*). The degree of fuzzification was chosen as m = 1.5 since larger or smaller values did not improve the efficiency of the classification measured by AUC of ROC. The two methods yielded very similar classifications and classification probabilities (shown graphically in Fig. 6), given the high ROC AUC values comparing the two approaches.

### Statistical analysis

Since vestibular perceptual thresholds, including roll tilt thresholds, have been described to follow a lognormal distribution^73^, they were plotted as geometric means (c.f., Fig. 1), standard parametric statistical tests (analysis of variance, t-tests) were performed with the thresholds in log-units, and lognormal distributions were confirmed using Kolmogorov-Smirnov tests. Standard non-parametric tests (Mann-Whitney) were used when distributions were not normal or log-normal. P-values of 0.05 or less were accepted as significant and all tests were corrected for repeated measures.

De-identified data that were used in this study are available on the laboratory’s webpage: www.masseyeandear.org/research/otolaryngology/investigators/laboratories/jenks-vestibular-physiology-laboratory.
